# Circulating homocysteine and folate concentrations and risk of type 2 diabetes: A retrospective observational study in Chinese adults and a Mendelian randomization analysis

**DOI:** 10.3389/fcvm.2022.978998

**Published:** 2022-11-14

**Authors:** Yating Cheng, Chen Wang, Xiaokang Zhang, Yue Zhao, Bingyu Jin, Chunfang Wang, Zhibing Lu, Fang Zheng

**Affiliations:** ^1^Center for Gene Diagnosis and Department of Laboratory Medicine, Zhongnan Hospital of Wuhan University, Wuhan, China; ^2^Laboratory Medical Center, Hospital of Youjiang Medical University for Nationalities, Baise, China; ^3^Department of Cardiology, Zhongnan Hospital of Wuhan University, Wuhan, China; ^4^Institute of Myocardial Injury and Repair, Zhongnan Hospital of Wuhan University, Wuhan, China

**Keywords:** homocysteine, folate, type 2 diabetes, retrospective study, Mendelian randomization

## Abstract

**Background:**

The relation between circulating homocysteine (hcy) and folate concentrations and risk of type 2 diabetes mellitus (T2DM) has been evaluated in several observational studies with inconsistent results; and it is unclear about their causal relationships. Our aim was to assess the causality association between circulating hcy or folate concentrations and the development of T2DM using Mendelian randomization (MR) analysis, based on results of an observational study in Chinese adults.

**Methods:**

We conducted an observational study of 370 patients with T2DM and 402 controls after routine physical examination who consulted at the Zhongnan Hospital of Wuhan University between March 2021 and December 2021. Correlations between hcy and folate and the incidence of T2DM were quantified using logistic regression models. Two-sample MR analysis was conducted using summary statistics of genetic variants gained from 2 genome-wide association studies (GWAS) on circulating hcy and folate concentrations in individuals of European ancestry and from an independent GWAS study based on DIAMANTE meta-analysis.

**Results:**

In the observational study, after logistic regression with multiple adjustment, lower hcy and higher folate levels were identified to be associated with the risk of T2DM, with OR (95% CI) for hcy of 1.032 (1.003–1.060); while 0.909 (0.840–0.983) for folate. In the MR analysis, the OR for T2DM was 1.08 (95% CI: 0.95, 1.21; *P* = 0.249) for each SD unit increase in genetically predicted homocysteinemia and the OR for T2DM per SD increase in genetically predicted folate elevation was 0.80 (95% CI: 0.60, 1.00, *P* = 0.026).

**Conclusions:**

We discovered that high circulating hcy and low folate concentrations were related with an increased risk of developing T2DM in Chinese adults. Moreover, MR analysis provided genetic evidence for a possible causal relationship between serum folate and the risk of T2DM.

## Introduction

**Figure 1:**
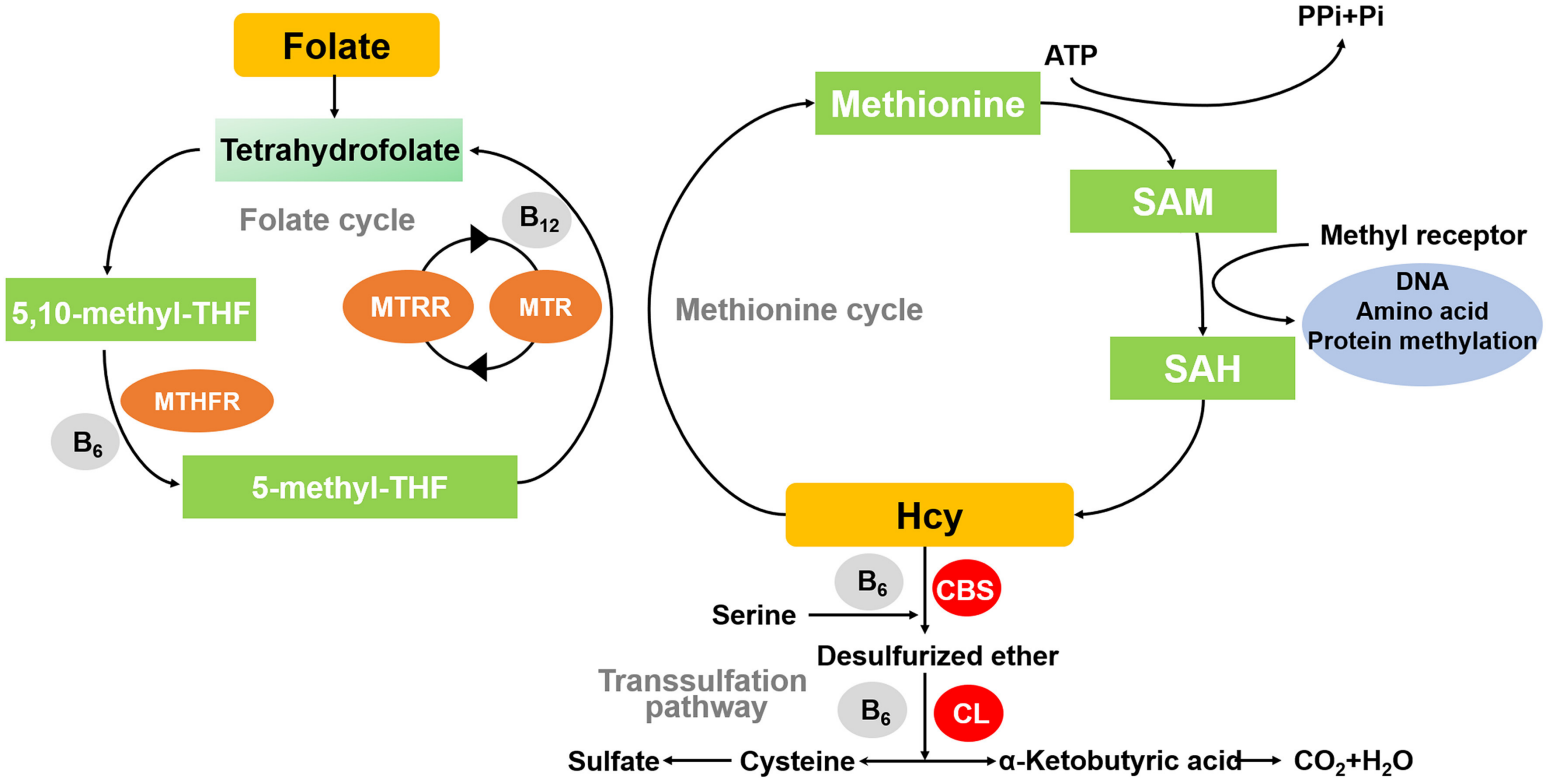
Diagram of methionine cycle. MTHFR, methylene tetrahydrofolate reductase; MTR, 5-methylthioribose; MTRR, Methionine synthase reductase; CBS, Cystathione-β-synthase; CL, Cystathione-β-cleavage enzyme; SAH, S-adenosyl-homocysteine; SAM, S-adenosyl methionine.

The metabolism of homocysteine (hcy) and folate has important roles in nucleic acid synthesis, amino acid homeostasis, epigenetic maintenance, redox defense, and methylation (1). This metabolic process is also known as the methionine cycle. In this process, methionine interacts with ATP to produce S-adenosylmethionine (SAM) catalyzed by methionine adenosyltransferase. SAM is methylated by transferring methyl to another substance catalyzed by methyltransferase, and SAM is changed to S-adenosylhomocysteine (SAH), which is deadenosylated to produce hcy (2). In the presence of methionine deficiency, hcy undergoes remethylation. This metabolic pathway requires folate as a donor of methyl groups to restore methionine. Remethylation is catalyzed by methionine synthase (MS) with vitamin B12 as cofactor and 5-methyltetrahydrofolate (5-MTHF) as methyl donor to transfer methyl from 5-methyltetrahydrofuran to hcy, resulting in the formation of new methionine, which can be used for protein synthesis or reconverted to SAH (3). If the amount of methionine is high enough, a transsulfuration reaction occurs. The enzyme critical in this metabolic pathway of hcy is Cystathione-β-synthase (CBS), an enzyme that requires vitamin B6 as a cofactor to catalyze the reaction of serine with hcy to form cystathionine (4). If the remethylation and/or transsulfuration pathways are impaired, hcy will accumulate in the cells and there will be the development of hyperhomocysteinemia (Figure 1).

Deficiency of folate contributes to elevated hcy concentrations, which have been demonstrated to be a risk factor for the development of cardiovascular disease and T2DM (5–7). This association is explained by several possible underlying pathophysiological mechanisms, such as the adverse effects of acute and prolonged exposure to high hcy concentrations on the cell viability of pancreatic β-cells (8–10). The generation of ROS by hcy in the redox-cycling reaction leads to a decrease in the viability of insulin-secreting cells, which in turn results in diminished glucokinase phosphorylation, weakened insulin-secreting response and cell death (11, 12), which are essential components of the pathogenesis of T2DM (13, 14). However, there are inconsistent relationships in epidemiological studies regarding the association between hcy concentrations and the incidence of T2DM (10, 15–18). It was the same as that contradictory conclusions have been reported about whether folate supplementation can diminish the incidence of diabetes (19–21).

Given these conflicting results, it is necessitated to investigate the causal association between blood homocysteine and folate levels and the incidence of T2DM. Nevertheless, observational researches are prone to reverse causality or to be influenced by confounding factors. Therefore, more endeavor is required to obviate the interference factors. Mendelian randomization (MR) analysis is a valuable approach that employs genetic variants, usually single nucleotide polymorphisms (SNPs), as instrumental variables (IVs) to evaluate the causal relationship between exposure and outcome (22). According to Mendel's second law, alleles are randomly assigned to offspring gametes at gamete formation and genotypes are already fixed before the onset of disease, so causal estimates of MR are minimally affected by reverse causality or confounding effects (23).

Previous MR reports on the causal effect of hcy on T2DM presented controversial results, and there were no MR studies of the causal impact of folate on T2DM before. Consequently, we performed an observational study in Chinese adults to investigate the associations between hcy, folate and T2DM, together with a two-sample MR analysis to further explore the causal relationships between circulating hcy or folate levels and T2DM.

## Methods and materials

### Study population

We conducted a retrospective observational study with 370 type 2 diabetic patients and 402 people after routine physical examination who consulted at Zhongnan Hospital of Wuhan University between March 2021 and December 2021. Their residual blood was collected after the clinical examination. The diagnosis of type 2 diabetes is based on WHO diagnostic criteria for diabetes, which include fasting blood glucose value ≥ 7.0 mmol/L, random blood glucose value ≥ 11.1 mmol/L, glucose load ≥ 11.1 mmol/L after 2 h, and accompanied by clinical symptoms of insulin resistance. None of the participants had a diagnosis of cancer, acute inflammation, blood system disease, congenital heart disease, previous myocardial infarction (MI), liver or kidney failure, or other serious illnesses. Our study protocol was approved by the Ethics Committee of Zhongnan Hospital of Wuhan University (Approval No.: 2020195). Flow diagram of the study design is given in Supplementary Figure 1.

### Laboratory examinations

Peripheral venous blood was collected from all participants after an overnight (8–10 h) fast. Serum folate was quantified by the Chemiluminescence method (Supplementary material). The fasting level of homocysteine was also measured using enzyme cycle method (Supplementary material). Quality control samples were scattered throughout each analysis. On the basis of the measurements of these quality control samples, the mean intra- and inter-assay coefficient of variation (CVs) were ≤8.33 and ≤12.5% for folate and ≤5.0 and ≤5.9% for homocysteine, respectively. Other biochemical markers such as total cholesterol, triglycerides, HDL cholesterol, LDL cholesterol, and fasting glucose were measured by enzymatic methods completed at Beckman coulter AU5800. Superoxide dismutase (SOD) was measured by the colorimetric method. Vitamin B12 and vitamin B6 were measured by the chemiluminescence method.

### Data sources for MR analysis

T2DM population was obtained from the DIAMANTE (European) meta-analysis from the Diabetes Genetics Replication and Meta-analysis (DIAGRAM) consortium (24), a meta-analysis of 32 GWAS with 74,124 T2DM cases (mean age ~63 years old) and 824,006 controls (mean age ~54 years old) of European descent, adjusted for study-specific covariates and corrected for the genomic-control.

The genetic variants associated with circulating hcy and folate were derived from two previous genome-wide association studies (GWAS) meta-analyses including 44,147 and 37,465 individuals of European ancestry, respectively (25, 26). Single nucleotide polymorphisms (SNPs) that reached the genome-wide significance threshold (*p* < 5 × 10^−8^) and without linkage disequilibrium (r^2^ < 0.1 in the 1000 G European population) for the exposure phenotype were used as instrumental variables (Supplementary Table 1). And the information of SNPs in the outcome data was shown in Supplementary Table 2. The variation in homocysteine and folate concentrations explained by SNPs was estimated to be 6.0 and 1.0%, correspondingly (25, 26). In addition, we examined the secondary phenotypes of each SNPs by PhenoScanner (27) and found four SNPs associated with homocysteine, including rs548987, rs2251468, rs838133, and rs1047891, correlated with other traits (28), and no folate-related SNPs were identified to be related to other phenotypes.

### Statistical analysis

The basic information and clinical characteristics of continuous variables with normal distribution were described by mean ± standard deviation (SD). The median and inter-quartile range described the continuous variables of abnormal distribution. Student's *t* test or Mann-Whitney *U* tests were applied to compare the continuous variables between the two groups depending on the distribution and chi-square test for the categorical variables. Conditional logistic regression models were utilized to investigate the relationship between circulating hcy, folate concentrations and the risk of T2DM. Factors such as age, sex, body mass index (BMI), fasting glucose, total cholesterol, triglycerides, HDL cholesterol, LDL cholesterol, history of smoking and drinking, and hypertension were adjusted sequentially.

Furthermore, the potential non-linear associations between circulating hcy or folate and T2DM were evaluated on a continuous scale with restricted cubic spline curves based on the logistic regression model (29). In this analysis, the number of knots corresponding to the minimum Akaike Information Criterion (AIC) was screened based on the AIC guidelines, and the reference values were set according to the normal clinical reference ranges of hcy and folate concentrations. In addition, we conducted a stratified analysis depending on the distribution of the variables using an unconditional logistic regression model, corrected by adjusting other covariates, and further tested the meaning of the interaction modification by incorporating a multiplicative interaction term to the model.

For MR analysis, fixed-effects inverse variance weighted method (IVW) was used as the main analysis method to obtain the combined causal effect of each IV, which was supplemented with weighted median and MR-Egger methods for multiple validation because the IVW may be biased by pleiotropic effects (30). Additional analyses excluded four SNPs with secondary phenotypes associated with hcy to preclude potential pleiotropic effects. Heterogeneity was evaluated employing the Q statistic in the IVW method, and *P*-value < 0.05 was considered significant. The test for pleiotropy was determined on MR-Egger regression to find the *P-*value of the intercept (31). The odds ratios (ORs) and corresponding 95% confidence intervals (CIs) for T2DM were calculated as a ratio of each standard deviation (SD) increase in genetically predicted circulating hcy and folate concentrations.

All statistical analyses were conducted through GraphPad Prism 8.0 (GraphPad Inc., United States) and R Software 4.0.2. The threshold of *P*-value on both sides was set to 0.05, which was statistically significant.

## Results

### There were observational relationships between circulating homocysteine or folate concentration and risk of T2DM

Consistent with previous studies, statistically significant differences existed between the T2DM and control groups with regard to identified diabetes risk factors, such as BMI, history of hypertension, fasting glucose, blood lipids and SOD (Table 1). The results of vitamin B12 and vitamin B6 were displayed in Supplementary Table 3. Median homocysteine concentrations were noticeably higher (*P* = 0.002) and median folate levels were obviously lower (*P* < 0.0001) in the T2DM group when compared to the control group.

**Table 1:** Characteristics in controls and patients with type 2 diabetes.

**Characteristics**	**NC (*n =* 402)**	**T2DM (*n =* 370)**	***P*** **value**
Age (years)	52.45 ± 8.97	68.25 ± 13.16	**< 0.0001**
Men (no, %)	259 (64.43)	220 (59.46)	0.1592
Women (no, %)	143 (35.57)	150 (40.54)	
Height (cm)	168.6 ± 7.36	167.9 ± 7.28	0.1990
Weight (kg)	67.54 ± 10.80	71.03 ± 10.33	**< 0.0001**
BMI (kg/m^2^)	23.74 (21.82, 25.41)	25.60 (23.07, 27.40)	**< 0.0001**
Hypertension history (no, %)	97 (24.13)	174 (47.03)	**< 0.0001**
Smoking history (no, %)	163 (40.55)	171 (46.22)	0.1268
Drinking history (no, %)	169 (42.04)	181 (48.92)	0.0600
Diabetic complications (no, %)	0 (0.00)	165 (44.59)	**< 0.0001**
FPG (mmol/L)	5.27 (5.06, 5.88)	6.96 (5.60, 8.77)	**< 0.0001**
TC (mmol/L)	5.05 (4.30, 5.31)	5.18 (4.26, 5.62)	0.0899
TG (mmol/L)	1.29 (1.00, 1.75)	1.78 (1.23, 2.31)	**< 0.0001**
HDL-C (mmol/L)	1.19 (1.11, 1.43)	0.94 (0.78, 1.15)	**< 0.0001**
LDL-C (mmol/L)	2.80 (2.41, 3.45)	3.18 (2.58, 3.72)	**0.0003**
SOD (U/ml) (50vs50)	177.80 ± 16.18	110.90 ± 27.87	**< 0.0001**
Homocysteine (μmol/L)	13.22 (10.79, 16.90)	14.99 (11.17,20.31)	**0.0002**
Folate (ng/mL)	7.47 (5.69, 10.21)	6.68 (5.04, 8.55)	**< 0.0001**

Data were shown as mean ± SD, median (25 percentiles, 75 percentiles). BMI, body mass index; FPG, fasting plasma glucose; TC, total cholesterol; TG, triglycerides; HDL-C, high-density lipoprotein cholesterol; LDL-C, low-density lipoprotein cholesterol; SOD, Super oxide dismutase. Entries in bold font indicate statistically significant (*P* < 0.05).

**Figure 2:**
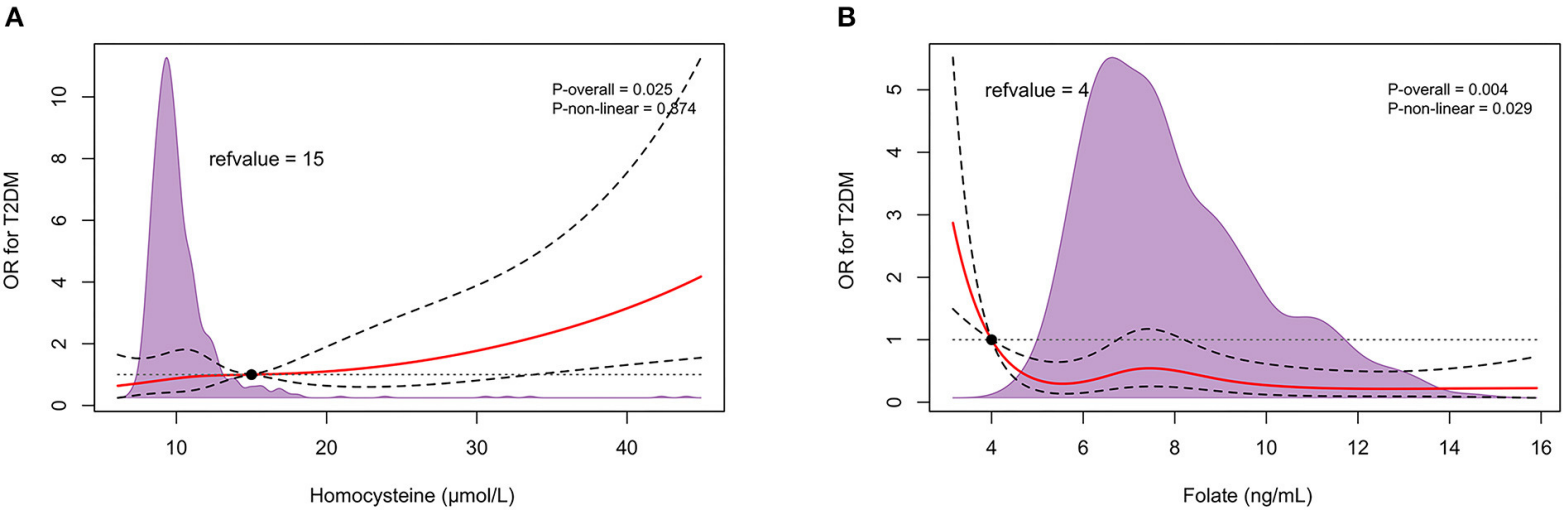
In retrospective observational study, restricted cubic spline relationship between circulating homocysteine **(A)**, folate **(B)** and T2DM onset. Red solid and black dashed lines depict ORs and 95% CIs for log-transformation of hcy, folate concentrations in conditional regression models based on restricted cubic splines. Reference values were set at 15 μmol/L and 4 ng/mL, respectively, depending on the clinical normal reference range. The purple shaded area represents the distribution density of hcy and folate in the total population (*n* = 772). The result has been adjusted for age; sex; body mass index; smoking history; drinking history; hypertension history; fasting total cholesterol, triglycerides, HDL cholesterol, LDL cholesterol, and glucose levels. T2DM, type 2 diabetes.

After sequentially adjusting for age, sex, and diabetes risk factors, ORs (95% CIs) for circulating homocysteine concentrations were 1.023 (1.005–1.043), 1.027 (1.007–1.050), and 1.032 (1.003–1.060), as well as ORs (95% CIs) for serum folate concentrations were 0.933 (0.881–0.987), 0.924 (0.869–0.982), and 0.909 (0.840–0.983), respectively, with significant differences in *P* values (Table 2). Restricted cubic spline curves revealed a forward linear relationship between circulating homocysteine and the incidence of T2DM (*P* = 0.025) (Figure 2A), while serum folate showed an inverse non-linear relationship with the occurrence of T2DM (*P* = 0.004) (Figure 2B).

**Table 2:** Adjusted ORs for T2DM incidence according to homocysteine and folate concentrations.

	**Homocysteine**		**Folate**	
	**OR (95% CI)**	***P*** **value**	**OR (95% CI)**	***P*** **value**
Model^a^	1.038 (1.020–1.058)	0.0001	0.886 (0.843–0.930)	< 0.0001
Model^a^	1.032 (1.003–1.060)	0.0247	0.909 (0.840–0.983)	0.0177

a Not adjusted.

b Adjusted for age (continuous variable), BMI (continuous variable), sex (male or female), Fasting plasma glucose (continuous variable), total cholesterol (continuous variable), triglycerides (continuous variable), HDL cholesterol (continuous variable), LDL cholesterol (continuous variable) and hypertension (yes or no).

**Figure 3:**
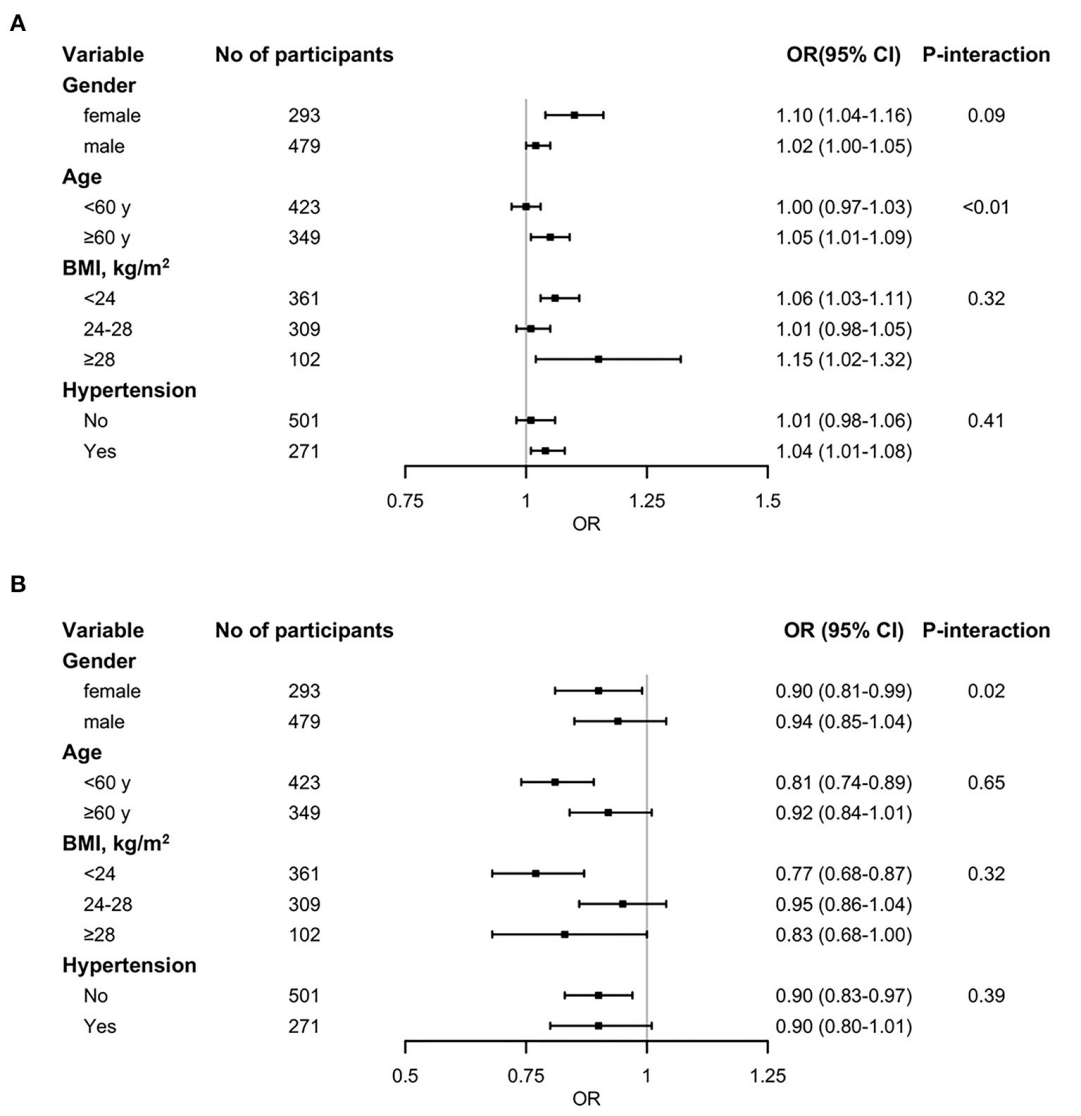
Stratified analysis of the association between circulating homocysteine **(A)** and folate **(B)** concentrations and T2DM. The black squares denote ORs for T2DM risk and the horizontal line signify 95% CI (*n* = 772). Models were adjusted for age, sex, body mass index, smoking history, drinking history, hypertension history, fasting total cholesterol, triglycerides, HDL cholesterol, LDL cholesterol, and glucose levels. P-interaction means the *P* value of the multiplicative interaction.

In the stratified analysis, we identified no meaningful difference of hcy and T2DM incidence between subgroups based on gender, BMI, and history of hypertension, but there were significant differences in the age categories (Figure 3A). A positive association was observed in those aged ≥60 years (OR: 1.05; 95% CI: 1.01, 1.09), but not in participants aged < 60 years (OR: 1.00; 95% CI: 0.97, 1.0.3). In addition, we also observed no variance of folate and the incidence of T2DM in age, BMI, or hypertensive subgroup analysis (Figure 3B). But in female, there was a negative correlation (OR: 0.90; 95% CI: 0.81, 0.99; P-interaction = 0.02), while not in male (OR: 0.90; 95% CI: 0.81, 0.99).

### A possible negative causal relationship between serum folate and the risk of T2DM was identified in MR analysis

**Figure 4:**
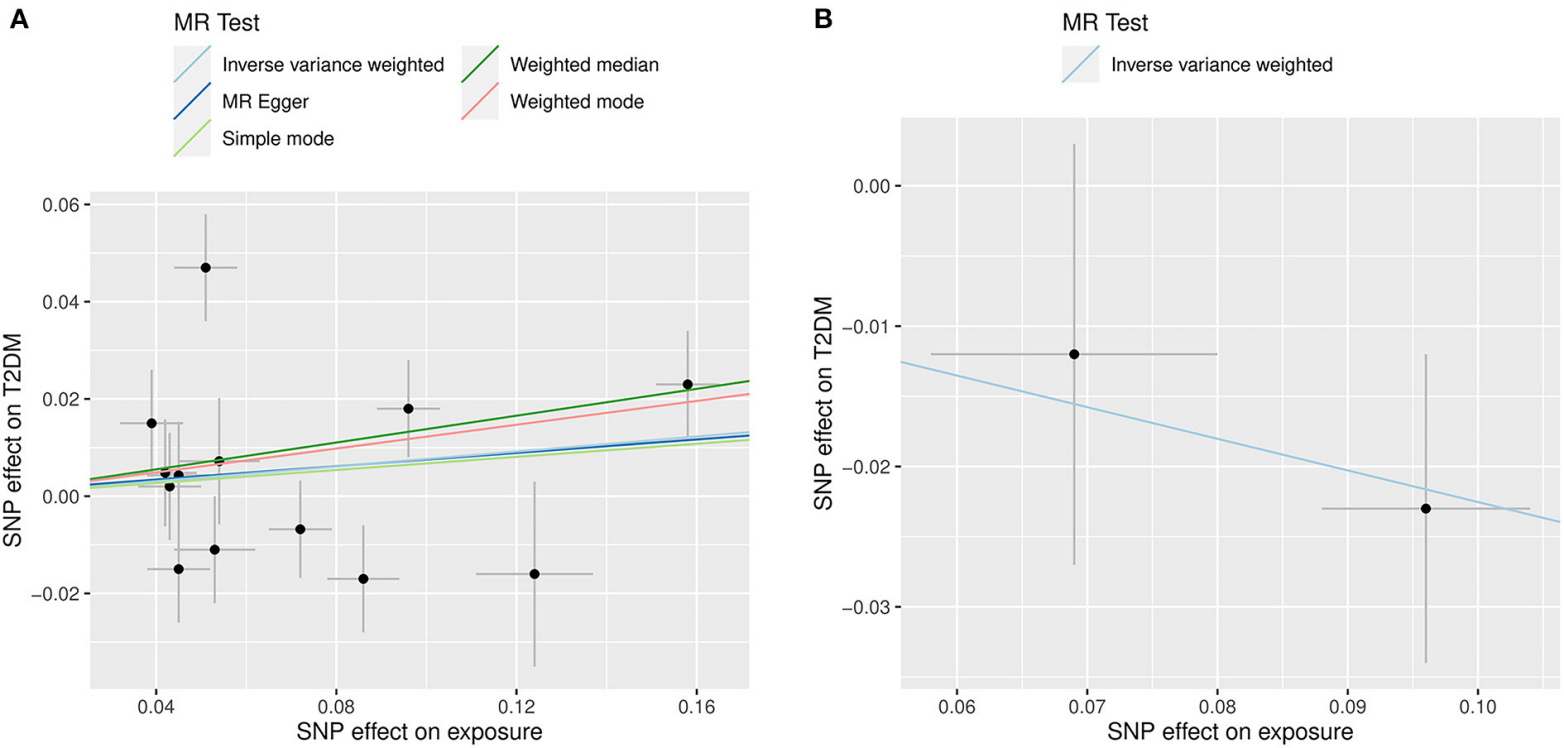
Scatter plots illustrating the results of the summary-level Mendelian randomization analysis. The x-axis presents the effect of exposure variables and the y-axis denotes the effect of outcome. The analysis was performed on 898,130 individuals of European ancestry from the DIAMANTE consortium. **(A)** Represents the effect of SNP on homocysteine levels (X-axis, per SD) and on T2DM [Y-axis, log (OR)], 95% CI; **(B)** Indicates the effect of SNP on folate levels (X-axis, per SD) and on T2DM [Y-axis, log (OR)], 95% CI.

The primary MR results indicated no relationship between genetically predicted circulating hcy levels and risk of T2DM, the OR for T2DM was 1.08 (95% CI: 0.95, 1.21; *P* = 0.249) for each SD unit increase in genetically predicted hcy concentrations. Similar results from the MR-Egger method were also consistent with this (OR: 1.07; 95% CI: 0.77, 1.38; *P* = 0.666). However, the weighted median method demonstrated a forward association between genetically predicted higher circulating hcy concentrations and for the risk of T2DM (OR: 1.15; 95% CI: 1.03, 1.26; *P* = 0.019) (Figure 4A, Table 3). These connections persisted after the exclusion of four pleiotropic SNPs (Supplementary Table 4). On the other hand, there was a suggestive association between higher genetically predicted folate levels and a lower risk of T2DM (OR_SD_: 0.80; 95% CI: 0.60, 1.00, *P* = 0.026) (Figure 4B, Table 3). Heterogeneity analysis displayed no heterogeneity in MR analysis (Supplementary Table 5) and the *P* value of MR-Egger intercept indicated no remarkable directional polymorphism (Supplementary Table 6).

**Table 3:** Summary Mendelian randomization estimates of circulating homocysteine and folate levels on risk of type 2 diabetes.

**Outcome**	**Exposure**	**Method**	**b**	**se**	**P**	**or**	**or_lci95**	**or_uci95**
Type 2 diabetes	Homocysteine	Inverse variance weighted	0.077	0.066	0.249	1.08	0.95	1.21
Type 2 diabetes	Homocysteine	MR Egger	0.069	0.155	0.666	1.07	0.77	1.38
Type 2 diabetes	Homocysteine	Weighted median	0.138	0.059	**0.019**	**1.15**	**1.03**	**1.26**
Type 2 diabetes	Homocysteine	Weighted mode	0.122	0.062	0.072	1.13	1.01	1.25
Type 2 diabetes	Homocysteine	Simple mode	0.067	0.117	0.576	1.07	0.84	1.30
Type 2 diabetes	Folate	Inverse variance weighted	−0.225	0.101	**0.026**	**0.80**	**0.60**	**1.00**

b, beta; se, standard error; or, odds ratio; P, *P* value; ci, confidence interval; lci95, uci95. Entries in bold font indicate statistically significant *p* < 0.05.

## Discussion

In this retrospective observational study, we observed a positive trend between circulating homocysteine concentrations but a negative correlation between folate levels and the risk of T2DM. And MR analysis results supported the causal relationship between folate and T2DM. Nonetheless, there was no causal relationship between hcy concentrations and T2DM risk. In addition, in the stratified analysis, we found that the effect of hcy on T2DM was more pronounced in individuals aged ≥ 60 years, while the protective effect of folate was more prominent in women.

Several observational studies have discovered hyperhomocysteinemia as a risk factor for T2DM and related complications (32–34). A prospective cohort study has shown that elevated hcy levels were associated with increased cardiovascular risk, particularly in patients with diabetes (35). In a meta-analysis of 4011 patients with T2DM and 4303 normal controls, absolute pooled mean concentrations of hcy were detected to be appreciably higher in patients with T2DM, and the estimated causal OR associated with T2DM was 1.29 for 5 μmol/L increment in hcy, providing strong evidence for the relationship between hcy levels and the development of T2DM (36). Nevertheless, two-sample MR analysis in our study did not provide genetic evidence to support a causal relationship between hcy levels and T2DM risk. Consistent with our MR results, in the Prospective Investigation of the Vasculature in the Elderly in Uppsala (PIVUS) cohort, the authors showed no evidence of a causal relationship between hcy levels and fasting glucose, fasting insulin, or T2DM (37). This difference may also indicate that hcy maybe a risk marker for T2DM rather than a causal risk factor. Notably, we found a protective effect of genetically predicted folate levels on the risk of T2DM, which further indicate the association in the observational study was causal. Several researches of folate supplementation reducing the incidence of T2DM also support this causal relationship (20, 21, 38). A clinical trial that included 6,185 diabetic patients found that folate supplementation in T2DM patients resulted in lower hcy levels and better glycemic control (39). Lind MV et al. evaluated the effects of folate supplements on glucose metabolism and the risk of type 2 diabetes through a systematic review and meta-analysis of 29 randomized controlled trials involving 22,250 participants, finding when compared with placebo, folate supplementation lowered fasting insulin (WMD: −13.47 pmol/L; 95% CI: −21.41, −5.53 pmol/L; *P* < 0.001), and homeostasis model assessment for insulin resistance (HOMA-IR) (WMD: −0.57 units; 95% CI: −0.76, −0.37 units; *P* < 0.0001) suggests that folate supplementation may benefit glycemic homeostasis and hypoglycemia (40).

As for the underlying mechanisms, we speculated that hyperhomocysteinemia might cause oxidative stress, and the increased reactive oxygen species act as functional signaling molecules to activate various stress-sensitive signaling pathways, eventually lead to insulin resistance (41). It has been reported that homocystein(hcy) reduces the antioxidant capacity of the body by inhibiting glutathione production and suppressing the expression of Glutathione peroxidase (GSH-Px) and SOD (42). This is consistent with the lower SOD levels in T2DM patients compared with controls in our study. However, folate acts as a methyl donor in the methionine cycle and reduces hcy accumulation thus exerting its antioxidant properties, promoting the remethylation of homocystein to form methionine. Reports have also suggested that folate may exert antioxidant effects independent of hcy reduction by inhibiting NADPH oxidase-mediated superoxide anion production (43), and blocking superoxide production at the level of the mitochondrial electron transport chain (44) on vascular endothelium. Further studies are needed to better elucidate the relationship between hcy and folate and the pathogenesis of T2DM.

However, the controversial findings have also been reported in other studies (15, 19, 45). One probable explanation is that the sample sizes of these studies may not be large enough to identify a true association. Furthermore, in observational studies, regression methods may not provide unbiased estimates of the true association when confounders are not observed due to their usually unknown or unmeasured characteristics, or when the number of confounders is too large.

The strength of our study was based on the correlation results of the retrospective study, and further causal relationships were explored using MR methods. Through the results of previous GWAS meta-analysis of total blood homocysteine and folate levels, multiple SNP combined analyses were applied as instrumental variables to explore the causal relationship with T2DM, whereas previous MR analyses used only a single SNP as an instrumental variable, which may have biased the results. Moreover, to the best of our knowledge, we are the first study to use MR methods to investigate folate levels and the risk of T2DM. Nevertheless, the following limitations are also worthy of attention. First, in the observational study, we only collected basic clinical information such as BMI and history of hypertension of the participants, while the effects of confounding factors such as pharmacological use, dietary intake, and exercise status were not fully considered. Second, the MR analysis was performed on individuals of European ancestry, which was not further validated in our retrospective study owing to condition limitations. Finally, we only examined the causal association of hcy and folate with T2DM from a genetic perspective, without considering the influence of potential environmental factors.

## Conclusions

In summary, we found there was a possible negative causal relationship between serum folate and T2DM incidence but a positive correlation between homocysteine and T2DM in middle-aged and elderly Chinese.

## Data availability statement

The original contributions presented in the study are included in the article/Supplementary material, further inquiries can be directed to the corresponding author.

## Ethics statement

The studies involving human participants were reviewed and approved by the Ethics Committee of Zhongnan Hospital of Wuhan University. Written informed consent for participation was not required for this study in accordance with the national legislation and the institutional requirements.

## Author contributions

YC and FZ: designed research, conducted research, and wrote original draft. CheW and XZ: conducted research, analyzed data, and performed statistical analysis. YZ and BJ: analyzed data and performed statistical analysis. ChuW and ZL: supervised and edited. FZ: supervised, edited, and secured funding. All authors contributed to the article and approved the submitted version.

## Funding

This research was supported by the National Natural Science Foundation of China (Grant Nos. 81871722 and 82072373); Translation Medicine and Interdisciplinary Research Joint Fund of Zhongnan Hospital of Wuhan University (Grant Nos. ZNLH201907 and ZNJC201932); and Zhongnan Hospital of Wuhan University Science, Technology and Innovation Seed Fund (Grant Nos. znpy2019054 and znpy2019049).

## Conflict of interest

The authors declare that the research was conducted in the absence of any commercial or financial relationships that could be construed as a potential conflict of interest.

## Publisher's note

All claims expressed in this article are solely those of the authors and do not necessarily represent those of their affiliated organizations, or those of the publisher, the editors and the reviewers. Any product that may be evaluated in this article, or claim that may be made by its manufacturer, is not guaranteed or endorsed by the publisher.
