# Supplementary material for: Circulating homocysteine and folate concentrations and risk of type 2 diabetes: A retrospective observational study in Chinese adults and a Mendelian randomization analysis
Source: Front Cardiovasc Med. 2022 Nov 14;9:978998. doi: 10.3389/fcvm.2022.978998 (PMC9726538; doi:10.3389/fcvm.2022.978998)
Supplement: Supplementary file 1 [file Data_Sheet_1.PDF]

Supplementary Material

1 Supplementary Figures

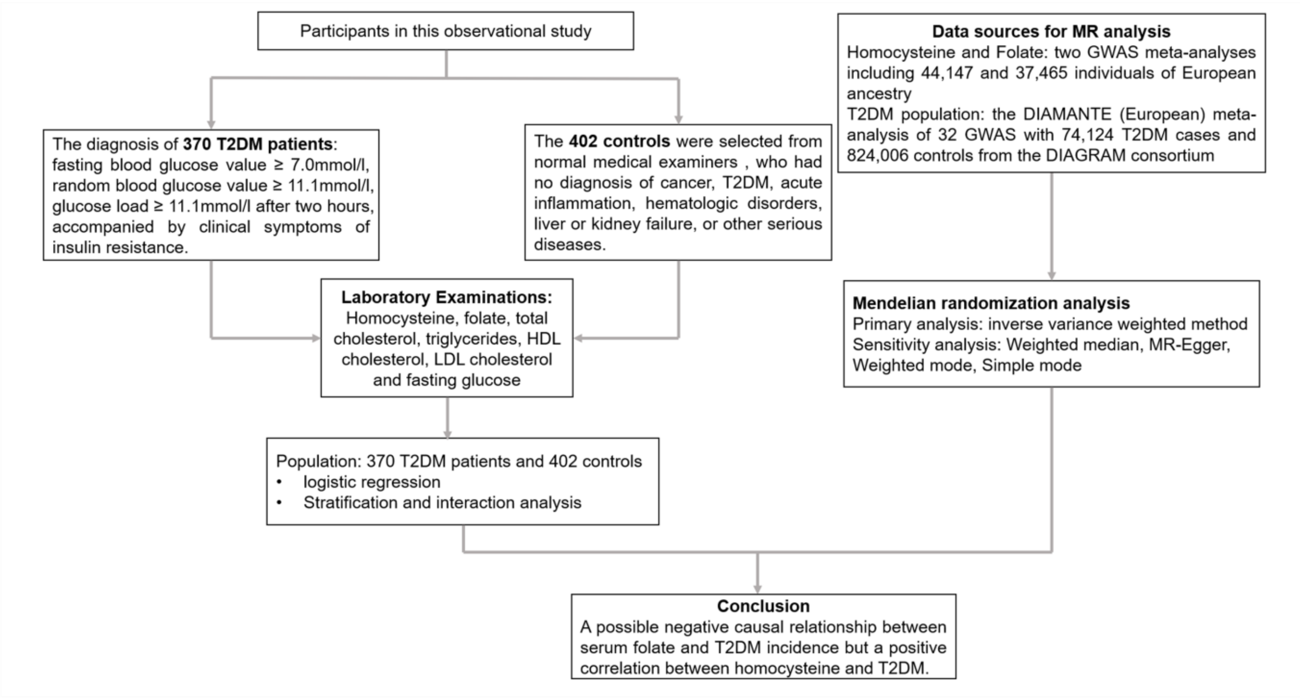

Supplementary Figure 1. Flow diagram of the study design.

2 Supplementary Tables

Supplementary Table 1. Information on instrumental variables.

Exposure	SNP	Beta	SE	Effect allele	non-effect allele	Effect allele freq	P value
Homocysteine	rs1801133	0.158	0.007	A	G	0.34	4.30E-104
Homocysteine	rs2275565	0.054	0.009	G	T	0.79	2.00E-10
Homocysteine	rs1047891	0.086	0.008	A	C	0.33	4.60E-27
Homocysteine	rs9369898	0.045	0.007	A	G	0.62	2.20E-10
Homocysteine	rs7130284	0.124	0.013	C	T	0.93	1.90E-20
Homocysteine	rs154657	0.096	0.007	A	G	0.47	1.70E-43
Homocysteine	rs234709	0.072	0.007	C	T	0.55	3.90E-24
Homocysteine	rs4660306	0.043	0.007	T	C	0.33	2.30E-09
Homocysteine	rs548987	0.06	0.01	C	G	0.13	1.10E-08
Homocysteine	rs42648	0.039	0.007	G	A	0.6	2.00E-08
Homocysteine	rs1801222	0.045	0.007	A	G	0.34	8.40E-10
Homocysteine	rs2251468	0.051	0.007	C	A	0.35	1.30E-12
Homocysteine	rs838133	0.042	0.007	A	G	0.45	7.50E-09
Homocysteine	rs12780845	0.053	0.009	A	G	0.65	7.80E-10
Folate	rs652197	0.069	0.011	C	T	0.18	1.40E-12
Folate	rs1801133	0.096	0.008	G	A	0.67	9.50E-53
Folate	rs17421511	0.098	0.01	G	A	0.83	1.80E-15

SE, standard error; SNP, single nucleotide polymorphism.

Supplementary Table 2. Information of SNPs in the outcome data.

outcome	phenotype	SNP	beta	se	RISK_ALLELE	OTHER_ALLELE	P value
T2DM	T2DM	rs1801133	-0.023	0.011	G	A	3.80E-02
T2DM	T2DM	rs2275565	0.0072	0.013	G	T	5.70E-01
T2DM	T2DM	rs1047891	0.017	0.011	C	A	1.30E-01
T2DM	T2DM	rs9369898	-0.0043	0.011	G	A	6.90E-01
T2DM	T2DM	rs7130284	-0.016	0.019	C	T	4.10E-01
T2DM	T2DM	rs154657	-0.018	0.01	G	A	8.10E-02
T2DM	T2DM	rs234709	-0.0068	0.01	C	T	5.10E-01
T2DM	T2DM	rs4660306	0.002	0.011	T	C	8.50E-01
T2DM	T2DM	rs548987	-0.012	0.015	G	C	4.10E-01
T2DM	T2DM	rs42648	-0.015	0.011	A	G	1.60E-01
T2DM	T2DM	rs1801222	-0.015	0.011	A	G	1.50E-01
T2DM	T2DM	rs2251468	0.047	0.011	C	A	1.50E-05
T2DM	T2DM	rs838133	0.0048	0.011	A	G	6.50E-01
T2DM	T2DM	rs12780845	-0.011	0.011	A	G	3.20E-01
T2DM	T2DM	rs652197	-0.012	0.015	C	T	4.30E-01
T2DM	T2DM	rs1801133	-0.023	0.011	G	A	3.80E-02
T2DM	T2DM	rs17421511	0.0081	0.014	G	A	5.60E-01

SE, standard error; SNP, single nucleotide polymorphism.

Supplementary Table 3. Vitamin B12 and Vitamin B6 in controls and patients with type 2 diabetes.

Characteristics	NC (n=210)	T2DM (n=205)	P value
Vitamin B12 (pg/mL)	496.40 (467.20, 623.00)	426.30 (352.60, 481.70)	0.0009
Vitamin B6 (μmol/L)	21.90 (17.35, 30.54)	19.56 (16.8, 30.81)	0.7854

Supplementary Table 4. Summary Mendelian randomization estimates of circulating homocysteine levels on risk of type 2 diabetes after the exclusion of the four pleiotropic single nucleotide polymorphisms.

Outcome	Exposure	method	nsnp	b	se	P value	or	or_lci95	or_uci95
type 2 diabetes	Homocysteine	MR Egger	10	1.64E-01	1.09E-01	1.72E-01	1.18E+00	9.64E-01	1.39E+00
type 2 diabetes	Homocysteine	Weighted median	10	1.41E-01	5.85E-02	1.58E-02	1.15E+00	1.04E+00	1.27E+00
type 2 diabetes	Homocysteine	Inverse variance weighted	10	7.27E-02	5.06E-02	1.50E-01	1.08E+00	9.76E-01	1.17E+00
type 2 diabetes	Homocysteine	Simple mode	10	1.03E-01	1.30E-01	4.49E-01	1.11E+00	8.54E-01	1.36E+00
type 2 diabetes	Homocysteine	Weighted mode	10	1.45E-01	6.22E+06	4.51E-02	1.16E+00	-1.22E+07	1.22E+07

SNP: single nucleotide polymorphism, b: beta, se: standard error, or: odds ratio, ci: confidence interval.

Supplementary Table 5. Heterogeneity test of Mendelian randomization.

outcome	exposure	method	Q	df	P value
type 2 diabetes	Homocysteine	Inverse variance weighted	7.66E-02	6.64E-02	2.49E-01
type 2 diabetes	Folate	Inverse variance weighted	7.14E-02	1.00E+00	7.89E-01

df: degrees of freedom.

Supplementary Table 6. MR-Egger regression analyses on detecting directional pleiotropy of Mendelian randomization.

outcome	exposure	egger intercept	se	P value
type 2 diabetes	Homocysteine	0.000660533	0.01176841	0.9562466
type 2 diabetes	Folate	NA	NA	NA

se: standard error.

3 Supplementary Methods

3.1 Folate Assay (Chemiluminescence Assay)

The Chemiluminescence method for folate detection is a competitive binding assay. Folate binding protein, mouse anti-folate binding protein, folate-alkaline phosphatase conjugate and goat anti-mouse capture antibody are added to the reaction tube along with paramagnetic particles. The folate in the sample competes with the folate-alkaline phosphatase conjugate to bind a limited number of binding sites on the folate-binding protein. The resulting complex is then bound to the solid phase via the mouse anti-folate binding protein. After incubation, the material bound to the solid phase is attracted by magnetic fields, while the unbound material is removed by rinsing. The chemiluminescent substrate Lumi-Phos 530 is then added and the light intensity generated in the reaction is measured by a luminometer. The amount of light produced is inversely proportional to the folate concentration within the sample. The folate concentration is determined from the stored multi-point calibration curve.

3.2 Homocysteine Assay (Enzyme Cycle Method)

The oxidized homocysteine (hcy) is degraded to free hcy, which reacts with S-adenosyl homocysteine (SAM) catalyzed by homocysteine-methyltransferase to form methionine and S-adenosyl homocysteine (SAH). SAH is hydrolyzed by SAH hydrolase to form adenosine and hcy.The generated hcy enters the hcy methyltransferase catalyzed reaction to form a cyclic reaction. This cyclic reaction results in a significant amplification of the detection signal. The formed adenosine is immediately dehydrogenated to hypoxanthine and ammonia, which further reacts with NADH catalyzed by glutamate dehydrogenase, converting NADH to NAD. the decrease in absorbance due to the reduction of NADH is detected at 340 nm in proportion to the concentration of hcy in the sample.
